# A segurança está à mesa: segurança e soberania alimentar no Brasil

**DOI:** 10.1590/0102-311XPT126424

**Published:** 2024-09-23

**Authors:** Giovani Bonfim

**Affiliations:** 1 Universidade de Brasília, Brasília, Brasil.

Ao definir “*acabar com a fome, alcançar a segurança alimentar, melhorar a nutrição e promover a agricultura sustentável*” como o segundo dos Objetivos de Desenvolvimento Sustentável (ODS) ^1^, a Organização das Nações Unidas (ONU) chamou atenção para a urgência do problema da fome no mundo. Segundo o relatório de 2022 da Organização das Nações Unidas para a Alimentação e Agricultura (FAO) ^2^ para a América Latina, o custo de manter uma alimentação saudável aumentou na região, o acesso a alimentos está mais caro em comparação a outras regiões do mundo e a insegurança alimentar se agravou, principalmente após o período da pandemia da COVID-19. Diante desse cenário, é relevante discutir o problema da segurança alimentar.

No Brasil, segundo o Instituto Brasileiro de Geografia e Estatística (IBGE) ^3^, ao final de 2023, havia 27,6% dos domicílios pesquisados (correspondendo a 21,6 milhões de domicílios) em situação de insegurança alimentar. O percentual de domicílios em estado de insegurança caiu em relação à última pesquisa, realizada em 2018, que registrou 36,7% (28,7 milhões de domicílios). No entanto, ter quase um terço dos domicílios brasileiros nessa situação exige do governo brasileiro medidas de enfrentamento e a elaboração (e fortalecimento) de políticas públicas para aumentar a segurança alimentar.

Nesse sentido, em 2021, foi realizado, na Fundação Oswaldo Cruz (FIOCRUZ), o seminário *online O Brasil Depois da Pandemia - Alimentação e Nutrição: Perspectivas na Segurança e Soberania Alimentar*, organizado pela Iniciativa Saúde Amanhã. Um dos resultados do evento foi a publicação do livro objeto desta resenha ^4^. Essa coletânea, assim como o seminário, faz parte da Estratégia Fiocruz para a Agenda 2030, cujos objetivos são contribuir para o sistema de saúde brasileiro a partir da perspectiva dos ODS.

Organizado em sete capítulos, e contando com pesquisadores de diversas áreas, o livro demonstra como o problema da segurança alimentar é interdisciplinar e envolve desde a produção dos alimentos até a sua chegada à mesa da população. Os capítulos abordam as transformações da agricultura brasileira, sistemas agroalimentares, políticas públicas, desigualdades, variações do consumo alimentar e externalidades na produção de alimentos brasileira.

O capítulo inicial, escrito por John Wilkinson, explora o sistema agroalimentar global e brasileiro diante da nova fronteira tecnológica. O autor analisa a estrutura da cadeia produtiva de alimentos à luz das ondas de evolução tecnológica, incluindo a revolução verde e os transgênicos. Além disso, Wilkinson destaca como os movimentos sociais influenciaram a alimentação, criticando os padrões de produção e o consumo da agropecuária em massa. A atual onda tecnológica, foco do capítulo, é liderada por inovações que vão além do agronegócio tradicional, pois *startups* e capital de risco desempenham um papel fundamental na produção de alimentos. Enquanto os atores tradicionais da agropecuária mostraram baixa flexibilidade diante das mudanças, grupos varejistas se adaptaram às demandas e criticaram o modelo da grande agricultura, surgido no Brasil no século XX.

No segundo capítulo, os autores Georges Flexor, Karina Kato & Sérgio Pereira Leite exploram as mudanças na agricultura brasileira relacionadas à segurança alimentar e nutricional. O texto é dividido em três partes: análise do impacto da geopolítica global e do ciclo de *commodities* na segurança alimentar; discussão sobre preços e custos dos alimentos, considerando o uso das terras e o meio ambiente; e abordagem da relação entre o sistema agroalimentar, as estratégias de desenvolvimento e a segurança alimentar. Os autores destacam como a interdependência dos mercados domésticos e internacionais afeta a segurança alimentar no Brasil, com convergência de preços das *commodities* agrícolas e aumento da produção desses produtos, além de impactos ambientais a longo prazo.

O terceiro capítulo, escrito por Silvia Zimmermann & Nelson Giordano Delgado, aborda a formulação de políticas brasileiras relacionadas à segurança alimentar e nutricional. Conquistas alcançadas ao longo de anos foram posteriormente desmanteladas devido à crise institucional que afetou o Brasil a partir de 2016. Os autores investigam o processo de construção do Sistema Nacional de Segurança Alimentar e Nutricional (SISAN), considerando aspectos legais, estruturas governamentais e participação da sociedade civil. Três políticas específicas foram selecionadas para análise: o Programa de Aquisição de Alimentos (PAA), o Programa Nacional de Alimentação Escolar (PNAE) e o Programa Bolsa Família. São oferecidas reflexões instigantes sobre as possibilidades de construção de um cenário mais favorável à segurança alimentar no Brasil.

O quarto capítulo, escrito por Rosangela Alves Pereira, Edna Massae Yokoo & Marina Campos Araújo, discute a evolução da nutrição deficiente na população brasileira. As autoras utilizam dados de sete fontes de diferentes períodos (*Estudo Nacional da Despesa Familiar* - ENDEF 1974-1975; *Pesquisa Nacional de Saúde e Nutrição* - PNSN 1989; *Pesquisa Nacional de Demografia e Saúde* - PNDS 1996 e 2006; *Pesquisa de Orçamentos Familiares* - POF 2002-2003 e 2008-2009; *Estudo Nacional de Alimentação e Nutrição Infantil* 2019) para analisar mudanças estruturais na nutrição dos brasileiros; além de discutirem a sindemia global e fatores comuns, como sistemas alimentares e desigualdades regionais. Os problemas analisados incluem subnutrição, sobrepeso/obesidade e doenças crônicas não transmissíveis (DCNTs) relacionados aos hábitos alimentares. A pobreza está ligada à má nutrição. Fatores como escolaridade e gênero também influenciam a exposição a esses problemas. Segundo as autoras, a obesidade não significa excesso de nutrientes saudáveis, mas sim a baixa qualidade na alimentação.

Renato Maluf & Luciene Burlandy são os responsáveis pelo quinto capítulo, no qual são exploradas a segurança e a soberania alimentar, destacando desigualdades e problemas como obesidade e desnutrição. Propõe-se, assim, uma abordagem estrutural e política para enfrentar esses desafios, considerando a conexão entre saúde e meio ambiente na produção de alimentos. O Brasil enfrenta, segundo os autores, obstáculos econômicos, políticos e culturais para realizar o seu potencial de liderar uma mudança no paradigma da alimentação no país, bem como a complexidade da situação, que envolve diversos atores nos sistemas alimentares, exigindo coordenação pública e privada em diferentes escalas. O texto apresenta uma perspectiva que envolve a complexidade ligada aos diversos atores e dimensões a fim de compreender e abordar essas questões.

No capítulo seis, Rosely Sichieri, Eliseu Verly Jr. & Ilana Nogueira Bezerra utilizam dados da POF para analisar mudanças nos padrões de consumo alimentar entre 2002-2003 e 2017-2018, focando no período 2008-2009 a 2017-2018. Eles exploram os desafios de saúde pública relacionados à obesidade e DCNTs, estratificando a análise por idade, renda e região geográfica. Destaca-se a transição alimentar, em que alimentos ultraprocessados substituem os naturais, resultando em déficits nutricionais, mesmo com sobrepeso. O capítulo questiona o aumento da obesidade e DCNTs apesar da estabilidade no consumo de alimentos processados entre 2008-2009 e 2017-2018.

O sétimo e último capítulo, escrito por Gustavo Noronha, analisa os custos do uso de agrotóxicos e como os ODS estão ligados às externalidades. São examinados os impactos dos agrotóxicos na saúde, no meio ambiente e na economia, destacando que o Brasil lidera o consumo mundial dessas substâncias. Os danos incluem perda de fertilidade do solo, contaminação da água e riscos para trabalhadores rurais e consumidores. O autor explora também métodos para calcular os custos externos dessas externalidades negativas.

Embora os capítulos tenham sido escritos separadamente, é perceptível que a segurança alimentar atua como fio condutor, inter-relacionando as narrativas. Isso permite que o leitor se aprofunde nos temas e, ao mesmo tempo, se informe sobre as diferentes facetas das questões alimentares no Brasil. Apesar do flerte de alguns dos capítulos com a soberania alimentar, esse tema não é muito explorado. Ao nos determos sobre as causas profundas da insegurança alimentar, chegamos a questões que os estudos de soberania alimentar abordam de forma mais robusta, por exemplo o uso da terra, a autonomia sobre o que e como cultivar, para quais consumidores vender etc.

Os pesquisadores que buscam soluções para aumentar a segurança alimentar no Brasil (e na América Latina) encontrarão nessa obra perspectivas e informações importantes na construção do conhecimento, o que pode gerar qualidade e saúde à mesa da população.
